# Multiple Primary Lung Cancers in Surgical Patients: Revisiting Martini and Melamed 50 Years Later

**DOI:** 10.3390/cancers18142284

**Published:** 2026-07-16

**Authors:** Stephanie Tuminello, Brian Housman, Diane Hwang, Jayme Leschly, Jai Mehrotra-Varma, Angelo Zegarelli, Bishoy Yacoub, Storm Alexander, Apichat Tantraworasin, Emanuela Taioli, Raja M. Flores

**Affiliations:** 1 Department of Thoracic Surgery, Icahn School of Medicine at Mount Sinai, New York, NY 10029, USA; stephanie.tuminello@mountsinai.org (S.T.);; 2 Institute for Translational Epidemiology, Icahn School of Medicine at Mount Sinai, New York, NY 10029, USA; 3 Tisch Cancer Institute, Icahn School of Medicine at Mount Sinai, New York, NY 10029, USA; 4 Department of Surgery, Faculty of Medicine, Chiang Mai University, Chiang Mai 50200, Thailand; 5 Clinical Surgical Research Center, Clinical Epidemiology and Clinical Statistic Center, Faculty of Medicine, Chiang Mai University, Chiang Mai 50200, Thailand

**Keywords:** multiple primary lung cancer, intrapulmonary metastasis, recurrence, lung cancer surgery

## Abstract

**Background:** The Martini and Melamed criteria for diagnosing multiple primary lung cancers (MPLCs) have remained in use for half a century despite substantial changes in lung cancer epidemiology, risk factors, imaging technologies, and treatment approaches. Correspondingly, the landscape of MPLC has likely changed, necessitating revisions to their diagnostic guidelines. **Research Question:** What are the clinicopathologic profiles of modern-day MPLC patients, and can these features be used to identify a subset of patients classified as MPLC that may warrant additional investigation as intrapulmonary metastasis (IPM)? **Study Design and Methods:** We conducted a retrospective cohort study using electronic medical record data from patients who underwent multiple lung cancer resections at Mount Sinai Hospital. Patient demographics, tumor characteristics, and surgical treatments were compared with those reported in the original Martini and Melamed series. We then proposed a reclassification framework incorporating a CT-based assessment of synchronous versus metachronous tumors and contemporary clinicopathologic features documented in imaging and pathology reports. **Results:** Ninety-one patients underwent successive lung cancer resections. Among first nodules treated with surgical resection, 46% exhibited pathologic features suggestive of metastasis within the lung, including visceral pleural invasion (18%), vascular invasion (24%), lymphatic invasion (36%), and/or lymph node positivity (3%). In contrast, among second nodules (second temporal surgery), 45% demonstrated ground-glass opacity (GGO) supportive of a diagnosis of MPLC. Using the original criteria, 83 patients (91%) were classified as having MPLC; this proportion decreased to 53 patients (58%) under the reclassification criteria. Regardless of the criteria applied, contemporary MPLCs were more likely to occur in older patients, women, those with adenocarcinoma histology, and treated with sublobar resections compared with MPLCs described in the Martini and Melamed era. **Conclusions:** MPLCs are increasingly recognized, and their clinical presentation has changed substantially over time. Modern imaging and pathologic assessment may be useful tools for diagnosing MPLCs, but this requires future validation. This has important implications for staging and treatment decision-making, as patients may be undertreated by not being offered multimodal treatments or over-treated with systemic therapy when surgery alone may be sufficient.

## Simple Summary

Lung cancers that appear in more than one area of the lung can either represent new independent cancers or spread from an original tumor. Distinguishing between these two situations is challenging but clinically important, as the diagnosis directly influences staging, treatment, and prognosis. The traditional Martini and Melamed criteria, developed decades ago, are still the most widely used framework for this distinction despite major advances in imaging, pathology, and our understanding of lung cancer biology. In this study, we examined whether the landscape of multiple primary lung cancers has changed over time. We also preliminarily explored how modern imaging and pathologic assessment may improve the diagnosis of multiple primary lung cancers, though future studies, including molecular validation, will be needed to determine the clinical utility of this modified diagnostic framework.

## 1. Introduction

In 1975, Martini and Melamed published their landmark paper outlining criteria for defining multiple primary lung cancer (MPLC) versus intrapulmonary metastases (IPM), where the initial lung cancer recurred [1]. Based on 50 patients, these criteria are still in use today. Yet, in the intervening half-century, the epidemiology of lung cancer patients has changed remarkably; adenocarcinomas, cancers among those with a never-smoking history, and females have all risen [2,3]. This is explained, at least partly, by the shift in frequencies of known risk factors for lung cancer; smoking and certain environmental exposures like asbestos are now less frequent, but average life expectancy, and thus cumulative exposure to carcinogens, has increased [3]. Notably, lung cancer remains the most frequently diagnosed cancer and the primary cause of cancer-related deaths worldwide, with about 2.5 million new cases and 1.8 million deaths per year, respectively [4].

The clinical management of lung cancer has also evolved in the past several decades. High-resolution chest imaging has improved, and low-dose computed tomography (LDCT) scans for early detection are now approved tools. These LDCT scans have a much higher resolution compared to chest X-rays, the original imaging technology used in Martini and Melamed, and can therefore detect more lung nodules, especially when the nodule size is small [5]. CT/PET scans can identify occult metastatic and nodal disease and are generally better than X-rays for visualizing ground-glass opacity in the lungs, an indicator of early cancer development [6]. This, together with the introduction of lung screening for high-risk individuals, has led to a stage shift towards stage I/II diagnoses of lung cancer [2] and correspondingly a greater number of lung cancer patients eligible to undergo surgical intervention, in parallel with much better outcomes and longer survival.

The capacity for lung cancer surgeries has also greatly expanded. The advent of video-assisted thoracic surgery (VATS) techniques, coupled with limited resections, now enables patients who, due to either age or comorbidities, would have been ineligible for traditional thoracic lobectomies to undergo surgery [7]. Limited resection means that more lung tissue, presumably healthy, remains postoperatively; however, remaining tissue can act as sites for future carcinogenic events. Patients who have undergone a limited surgical resection are at heightened risk of developing a second lung cancer, especially in the first year, but are at increased risk even 10 years out [8,9]. Long-term survivors, regardless of surgical type, may also be at heightened risk for subsequent lung cancer development [10]. Given these advancements, the number of individuals diagnosed with multiple lung cancers is expected to continue increasing. Therefore, there is an urgent need to be able to distinguish between cases of MPLC and IPM, and this has important implications for staging and treatment decision-making, as patients may be undertreated by not being offered multimodal treatments or overtreated with systemic therapy when surgery alone may be sufficient.

In the intervening 50 years since the publication of the Martini and Melamed paper, there has been much discussion about how best to adapt their criteria to the changing landscape of lung cancer. This includes a comprehensive histologic assessment to help differentiate between MPLC and IPM, largely based on comparing predominant and minor histologic subtypes, and cytologic and stromal features between tumors [11,12,13]. A recent publication from the International Association for the Study of Lung Cancer (IASLC) Pathology Committee echoed the importance of comprehensive histological evaluation coupled with radiologic findings [14]. They additionally noted the value of tumor molecular profiling [14]. Methodologies for performing this type of molecular diagnostics can range from simple targeted gene comparisons to comprehensive next-generation sequencing [14,15,16,17,18,19,20,21,22]. The expectation is that tumors representing MPLC will have substantial genomic heterogeneity, whereas tumors arising from IPM will be similar to the original cancer. As technology advances, some combination of histologic, radiologic, and molecular assessment will supplement what was originally proposed by Martini and Melamed [23], and in fact, such histomolecular algorithms have already been proposed [13]. However, there is complexity as to how best to utilize this type of genomic information. Accurately distinguishing MPLCs from IPMs requires discordance of trunk (or initiating) driver mutations, which would be indicative of a different clonal origin [16]. Differences in mutation profiles overall would be insufficient evidence, especially as both surgery and adjuvant systemic therapies can alter tumor mutation profiles through selective evolutionary pressure [24]. There is also the issue of limited generalizability. Despite being shown to be cost-effective in terms of quality-adjusted life years [25], tumor profiling for lung cancer is not automatic nor universally adopted by all institutions. Our team has recently shown that less than half of non-small cell lung cancer patients receive molecular diagnostic testing, and uptake varies along racial and socioeconomic lines [26]. To date, three major lung cancer research institutes, the Union for International Cancer Control (UICC), the American Joint Committee on Cancer (AJCC), and IASLC, have published guidelines for the management of MPLCs, based on Martini and Melamed, but without a clear consensus being reached for definitive, revised guidelines.

Here, we report on a large cohort of real-world clinical patients who underwent multiple surgeries for lung cancer treatment. We hypothesize that the substantial changes in lung cancer epidemiology, screening, and treatment that have occurred in the intervening years since Martini and Melamed are mimicked by a corresponding shift in the clinicopathologic profiles of MPLC patients today. Our initial aim was to describe this changing landscape of MPLCs in terms of sex, age, histology, and other clinicopathologic features. We additionally propose a clinicopathologic reclassification framework that incorporates data elements that were either unavailable or not recognized as clinically relevant during the Martini and Melamed era, with the goal of generating testable hypotheses for future validation and refinement of more robust diagnostic criteria.

## 2. Methods

### 2.1. Data Source & Patient Population

The Department of Thoracic Surgery maintains a prospective database detailing the demographics, pre-operative assessment, and surgical procedures of all thoracic surgery cases at the Mount Sinai Hospital. Additional information, such as from preoperative PET/CT scans and postoperative pathology reports, was queried from electronic medical records (EMRs). This research was approved by the Institutional Review Board (STUDY-15-01151) and received a waiver of informed consent.

**Figure 1:**
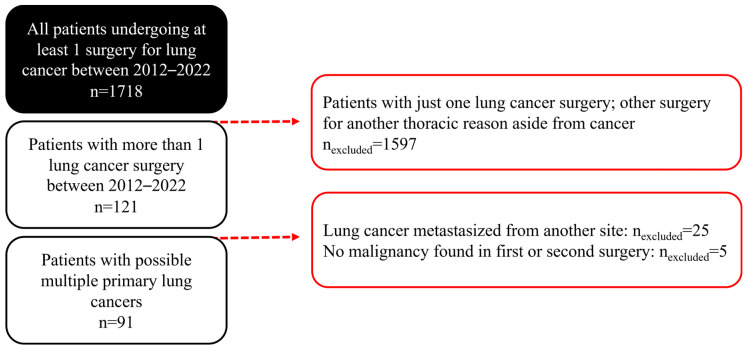
Patient Selection.

Between the years 2012 and 2022, 1718 patients had at least one surgery for lung cancer, 121 of whom had more than one lung cancer surgery. After excluding instances where the lung cancer was a metastasis from another organ site (for either the first or second lung cancer surgery) or where a nodule was ultimately determined to be benign, 91 patients underwent more than one surgery for possible MPLCs (Figure 1). The demographic and clinical profiles of this cohort are fully described in Supplementary Table S1. Cases with missing information required for classification were evaluated using all available clinicopathologic data. No imputation procedures were performed. Five of these patients had an additional third lung cancer surgery. However, given that the primary objective was to evaluate MPLC versus IPM classification consistency between the first and second surgically resected tumors, for patients undergoing more than two lung cancer resections, only the first and second tumors were included in the classification analysis. This approach was selected because the initial tumor pair typically represents the primary diagnostic challenge when distinguishing MPLC from IPM and most directly informs treatment planning and staging decisions.

### 2.2. Comparison of MPLCs, Now vs. Then

We first applied Martini and Melamed’s original criteria to the patient cohort. We compared their MPLCs with ours in terms of timeframe (synchronous vs. metachronous), demographics (sex, age at diagnosis), cancer characteristics (histology, site, features of intrapulmonary metastasis), and surgical treatment type. Variables were summarized as numbers, proportions, and means.

### 2.3. Preliminary Reclassification Framework for Diagnosing MPLCs

We next proposed an exploratory framework for diagnosing MPLC using clinicopathologic data readily available in the EMR data—specifically, to:

More accurately define the time between tumor nodules: Differentiating synchronous from metachronous nodules is key to distinguishing MPLCs from IPM. Synchronous disease was defined as tumors identified within 6 months of one another or when the subsequently resected nodule was retrospectively visible on imaging obtained at the time of the initial diagnosis. Metachronous disease was defined as a new lesion not visible on initial imaging and detected more than 6 months after the index tumor. The determination was based on a review of preoperative CT and PET/CT reports available in the EMR.

Incorporate observed ground glass opacity (GGO): GGO, characterized by tumor growth along preexisting alveolar structures [27,28], may indicate a distinct carcinogenic process [29]. Because GGO is commonly associated with adenocarcinoma in situ and a lepidic growth pattern and is often seen in primary lung adenocarcinomas, we considered this as supporting evidence of MPLC. Nodules were classified as GGO if CT reports closest to surgery used terms like “ground glass,” “non-solid,” “partially solid,” or “lepidic.” GGO in a second nodule supported an MPLC diagnosis. Information for GGO classification was extracted solely from radiology reports. Images were not re-reviewed by study investigators; GGO status was determined solely from the contemporaneous radiology interpretation.

Incorporate now-appreciated features of intrapulmonary metastasis: Key clinicopathologic factors were not well understood in 1975. For instance, it was not until 1992 that vascular or lymphatic invasion was first documented as a prognostic factor for non-small cell lung cancer (NSCLC) [29,30], with lymphovascular invasion now a well-characterized risk factor for distant metastases and death [31,32,33]. Visceral pleural invasion and the number of positive lymph nodes are also established risk factors for recurrence and survival [34,35]. The presence of any of these features at the first surgery was considered supportive of an IPM rather than an MPLC diagnosis.

GGO and pathologic features such as vascular invasion, lymphatic invasion, visceral pleural invasion, and nodal positivity were considered supportive clinicopathologic variables rather than definitive markers of clonality.

**Figure 2:**
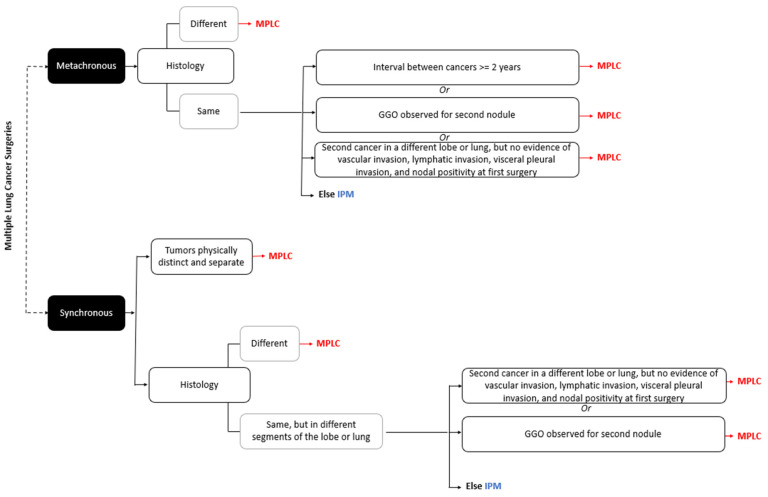
Proposed Clinicopathologic Reclassification Framework for Diagnosing MPLC vs. IPM.

Classification followed a hierarchical rule-based algorithm rather than a weighted scoring system. Consistent with the original Martini and Melamed framework, fulfillment of any qualifying MPLC criterion was considered sufficient for classification as MPLC. Clinicopathologic features associated with metastatic spread (vascular invasion, lymphatic invasion, visceral pleural invasion, and nodal positivity) were incorporated as supportive evidence of IPM but did not independently override qualifying MPLC criteria. Consequently, when MPLC-supporting and IPM-supporting features coexisted, classification was determined according to the predefined MPLC criteria. For example, tumors separated by a free interval ≥ 2 years were classified as MPLC regardless of the presence of vascular, lymphatic, pleural, or nodal involvement in the initial tumor. The revised framework is illustrated in Figure 2. Under the revised framework, tumors were classified as MPLC if any of the following criteria were met: (1) different histologic subtype; (2) metachronous presentation with a free interval ≥ 2 years; (3) presence of GGO in the second lesion; or (4) occurrence of the second lesion in a separate lobe or contralateral lung without evidence supporting pulmonary metastatic spread. Cases not meeting any MPLC criterion were classified as IPM. A comparison between this proposed, preliminary diagnostic framework and Martini and Melamed’s originally published criteria is provided in Supplementary Figure S1.

### 2.4. Statistical Analysis

Descriptive statistics were used to summarize the distribution of MPLC and IPM classifications, under Martini and Melamed’s original diagnostic criteria vs. our proposed preliminary reclassification framework. Categorical variables were reported as frequencies and percentages. The proportions classified as MPLC and IPM under each system were calculated with corresponding 95% confidence intervals (CIs). Agreement between classification systems was assessed using Cohen’s kappa (κ) with 95% CIs. McNemar’s test was used to evaluate differences in paired classifications between the two systems. All statistical tests were two-sided, with *p* < 0.05 considered statistically significant.

## 3. Results

### 3.1. MPLCs Using Original Martini and Melamed Criteria; Comparison with Martini and Melamed’s Identified MPLCs

Of the ninety-one patients treated with multiple surgeries for lung cancer, 83 (91%) met Martini and Melamed’s diagnostic criteria for MPLC. This left 8 (9%) cases which instead were considered instances of IPM. In their original manuscript, Martini & Melamed reported identifying 50 MPLCs [1].

Thirty-eight (46%) of our patients diagnosed with MPLC had synchronous nodules, whereas 45 (54%) had metachronous nodules under standard definitions. The mean time between the first and second lung cancer surgery was 30 months. In contrast, Martini and Melamed only reported 18 (36%) MPLCs that were found to be synchronous.

In terms of patient demographics, in our cohort, just 31 (37%) were male, whereas Martini and Melamed reported 38 (76%) male patients. The mean age at first lung cancer diagnosis was older, 67 years vs. 62 years for Martini and Melamed. Squamous histology was far less common (n = 6, 7%) and instead adenocarcinoma was more frequent (n = 73, 88%), while Martini and Melamed’s MPLCs were primarily squamous histology (n = 38, 76%). Having similar histology for both the first and second lung cancer occurred among the majority of our MPLCs: n = 71 (86%) vs. n = 33 (66%) of MPLCs identified by Martini and Melamed. They did not report the topographic site of their MPLCs, but for our cohort, right upper lobe (RUL) was the most common, n = 30 (36%). Ten (12%) MPLCs occurred in the same lobe. In terms of nodule size, the mean size was 1.96 cm for the first operative nodule and 1.84 cm for the second (Table 1).

Here, all patients underwent surgical resection (100%); for Martini and Melamed, n = 3 (6%) of MPLCs were not surgical cases. In terms of surgical approach, our MPLC patients differed substantially from those identified by Martini and Melamed. While 13 (26%) of their MPLC patients underwent pneumonectomies, none of our patients did so. Instead, among our MPLCs, sublobar resections were much more common: n = 55 (66%) vs. just n = 2 (4%) for Martini and Melamed (Table 1).

### 3.2. MPLCs Under a Proposed Clinicopathologic Reclassification Framework

We next incorporated CT and pathology data into the original MPLC criteria (Figure 1, Supplementary Figure S1) to create an exploratory revised framework. A total of 41/91 (45%) patients had second nodules with GGO features according to presurgical imaging, indicative of a second primary cancer. On the other hand, we considered visceral pleural invasion (n = 16, 18%), vascular invasion (n = 22, 24%), lymphatic invasion (n = 33, 36%), and lymph node positivity (n = 3, 3%) as supportive features of intrapulmonary metastasis; in total, 42 (46%) patients in our cohort had at least one of these (Supplementary Table S1).

Of the 91 patients who underwent multiple surgeries, 83 (91%; 95% Confidence Interval [CI]: 83.4–96.1%) were classified as MPLC under the original Martini and Melamed criteria, but only 53 (58%; 95% CI: 47.4–68.5%) met the revised definition (Table 2). Therefore, 33 patients were reclassified as IPM, while 3 patients went from IPM to MPLC. The full clinicopathologic description of these re-classified patients is available in Supplementary Table S2. MPLCs under the revised definition were still more likely to be metachronous (n = 40, 75%), be female (n = 35, 66%), be older (mean age 67 years), and have adenocarcinoma histology (n = 45, 85%) than historic Martini and Melamed-identified MPLCs (Table 1).

Agreement between classification systems was limited (Cohen’s κ = 0.084, 95% CI: −0.055 to 0.22). Despite an overall agreement of 60.4%, the proportion of cases classified as MPLC differed significantly between systems (McNemar’s test *p* < 0.001), with the revised criteria reclassifying a substantial proportion of cases previously designated as MPLC under the Martini and Melamed system to IPM.

## 4. Discussion

This manuscript critically reviews the limitations of the Martini and Melamed criteria for defining MPLC, given the many years and changes that have occurred since their seminal paper was published in 1975. By investigating a real-world surgical dataset, we observed that second primaries may be becoming more common, about 5% for all surgical lung cancer cases, compared to just 1% reported by Martini and Melamed. This increased prevalence may be explained, at least partly, by the improved postoperative survival of lung cancer patients, likely a reflection of better diagnostic discrimination, surgical techniques, earlier detection, or a combination thereof.

Additionally, we show here that the landscape of MPLCs has changed dramatically, mimicking the changing epidemiology of lung cancer overall. Patients with MPLC are now more commonly female, older, and have adenocarcinoma histology. These changes in the clinicopathological profiles of potential MPLCs may be clinically relevant. For instance, adenocarcinoma is associated with an increased risk of recurrent cancer compared to squamous cell carcinoma [36].

We next sought to investigate how clinicopathologic variables, nowadays readily available in EMR, may be useful for distinguishing between MPLC and IPM. First, by improving on delineating synchronous from metachronous cases. It was common (25/91) that the lung nodule treated during the patient’s second surgery was already present on the CT/PET scan performed prior to their first surgery. Comparably, only four of Martini and Melamed’s patients had two lesions already present on preoperative X-rays. This improved capability to identify synchronous cases can be attributed to the increased sensitivity of LDCT scans compared to X-rays. Chest X-rays can miss 30–50% of lung nodules smaller than 1–2 cm in diameter, whereas LDCT can detect nodules as small as 2–3 mm [36,37]. In fact, 8% of our observed nodules treated during the first surgery, and 12% of nodules treated during the second, were observed to be less than 1 cm.

The increased sensitivity of LDCT additionally allowed us to identify GGO. Because GGO is commonly associated with adenocarcinoma in situ and a lepidic growth pattern and is often seen in primary lung adenocarcinomas, we considered this as supportive of an MPLC diagnosis rather than IPM. Lastly, the oncological community increasingly recognizes visceral pleural invasion, vascular invasion, lymphatic invasion, and positive lymph nodes as key features of IPM. By including these clinicopathologic features in our proposed exploratory framework, 40% of our patient cohort was reclassified. The clinical implications of such reclassification extend beyond treatment selection alone. Classification as MPLC versus IPM directly influences TNM staging, as separate primary tumors are staged independently, whereas intrapulmonary metastases are incorporated into a single staging designation. Consequently, reclassification may result in substantial stage migration, altering prognosis estimates and eligibility for stage-specific treatment recommendations. Surgical decision-making may also be affected, as patients with presumed MPLC are often considered candidates for the curative-intent resection of multiple lesions, whereas identification of IPM may prompt greater consideration of multimodality treatment strategies incorporating systemic therapy and/or radiation. Reclassification may similarly influence recommendations for adjuvant treatment, particularly in patients whose revised staging places them into higher-risk categories. Follow-up strategies could also differ, as patients with IPM may warrant closer surveillance because of their increased risk of recurrence and disease progression. Finally, reclassification has important implications for the interpretation of survival outcomes in retrospective studies. The inclusion of patients with metastatic disease within MPLC cohorts may artificially worsen reported survival for MPLC, whereas the misclassification of true MPLC as IPM may obscure important differences in prognosis. Improved diagnostic classification is therefore essential not only for patient management but also for the accurate interpretation of clinical outcomes and future research studies. However, the framework proposed here for classifying MPLCs is preliminary, and the clinical utility of incorporating clinicopathologic features such as GGO, vascular invasion, lymphatic invasion, visceral pleural invasion, and lymph node positivity into definitions of MPLC requires future validation. While useful for generating testable hypotheses for future classification systems, it has not yet been validated and should not be considered as a new diagnostic standard at this time. This study also has several limitations inherent in its retrospective, single-center, surgical cohort design and the available clinical data sources, which are detailed below.

Given that this study relied on EMR data, it was difficult for us to assess how such reclassification translated into long-term clinical outcomes; thus, we acknowledge that the MPLC classification framework proposed here is preliminary and has not been validated as revised diagnostic criteria. Longitudinal analysis is a recognized limitation of using EMR data. In real-world settings, patients may move, change insurance, or switch healthcare providers and institutions [38]. Structured mortality data in the EMR typically exhibit sensitivity levels of around 65%, so approximately 35% of actual deaths are missed when relying on EMR data alone [39]. Another limitation of using EMR data is that a patient’s healthcare utilization pattern (visits or lack of visits) is often linked to their health state [38]; MPLCs in patients who changed clinical providers or died before their MPLC was diagnosed would not have been captured here. We did not incorporate autopsy findings, which could also represent an avenue of missed MPLC cases.

Nevertheless, by supplementing deaths captured in the EMR with obituary reports, it was possible to conduct a preliminary analysis of overall survival. Supplementary Figure S2 shows survival from first (Supplementary Figure S2A) and second surgeries (Supplementary Figure S2B) of patients classified as MPLC (n = 83) vs. IPM (n = 8) according to the traditional Martini and Melamed criteria (Hazard Ratio [HR]_first surgery_: 0.74; 95% CI: 0.21–2.33, *p* = 0.57; HR_second surgery_: 0.90; 95% CI: 0.27–2.98, *p* = 0.87). HRs and 95% CIs were derived from Cox proportional hazards regression, and *p*-values were calculated using the log-rank test. Supplementary Figure S3 shows survival from first (Supplementary Figure S3A) and second surgeries (Supplementary Figure S3B) of MPLC (n = 53) vs. IPM (n = 38) patients reclassified under our exploratory clinicopathologic criteria (HR_first surgery_: 0.67; 95% CI: 0.33–1.37, *p* = 0.27; HR_second surgery_: 1.05; 95% CI: 0.51–2.15, *p* = 0.89).

It should also be noted that the pathologic features of vascular invasion, lymphatic invasion, visceral pleural invasion, and lymph node positivity were abstracted from routine clinical pathology reports and were not independently verified through a central pathology review. As such, a limitation of the present study is that these data were not subject to standardized re-review by a dedicated pathologist, which may introduce variability in reporting practices. These features are not specific to intrapulmonary metastasis and may also be observed in aggressive primary lung tumors, nor are they definitive markers of clonality. These features should not be interpreted as binary diagnostic indicators in isolation but rather as supportive findings that should be interpreted in the broader clinicopathologic context when diagnosing MPLC. Having any potential pathway of metastatic invasion was marginally associated with worse overall survival (HR_first surgery_: 1.83; 95% CI: 0.89–3.76, *p* = 0.095, Supplementary Figure S4A). Among the individual pathologic features examined, only visceral pleural invasion was significantly associated with worse survival outcomes (HR_first surgery_: 4.67; 95% CI: 1.91–11.47, *p* = 0.001, Supplementary Figure S4C), whereas vascular invasion, lymphatic invasion, and lymph node positivity were not individually significant (*p* > 0.05, Supplementary Figure S4B,D,E). The presence of multiple metastatic pathways in the first surgically resected tumor was statistically significantly associated with impaired survival (HR_first surgery_: 1.52; 95% CI: 1.06–2.18, *p* < 0.001, Supplementary Figure S4F). Full survival analysis results, including HRs and 95% CIs, are reported in the Supplement. These findings suggest that evaluating these pathologic features in combination may provide greater prognostic value than assessing each feature independently. However, given the above-noted limitations regarding survival analysis in EMR, robust survival analysis, including disease-specific survival, was not possible, hindering our ability to assess whether our preliminary reclassification framework improves clinical discrimination. As several components incorporated into this revised classification framework, including GGO appearance, nodal involvement, and pleural invasion, are themselves closely linked to tumor biology and clinical prognosis, the reclassification presented here may inherently favor the classification of biologically indolent tumors as MPLC while categorizing more aggressive tumors as IPM. Further studies are needed to clarify the biological mechanisms through which these pathways may contribute to IPM. Further validation using independent cohorts and longitudinal clinical follow-up will be important to determine the extent to which the framework provides prognostic information beyond the biologic variables incorporated into the criteria.

The present study should be interpreted in the context of several other limitations. First, this was a retrospective single-center study with a relatively small cohort, which may limit generalizability and introduce selection bias. Although larger than the original M and M cohort, the modest sample size was further reduced following the application of the revised classification framework, resulting in smaller subgroup sizes. This may have limited statistical power to detect modest but clinically meaningful differences between groups, and estimates of reclassification frequency should therefore be interpreted with appropriate caution. In addition, due to the limited sample size and retrospective design, formal sensitivity analyses (e.g., alternative definitions of synchronous versus metachronous disease, exclusion of cases with missing data, or rule-based reclassification variations) were not performed, as such analyses would have resulted in unstable estimates and reduced interpretability. Future larger, multicenter studies would allow for more robust sensitivity and subgroup analyses to assess the stability of the proposed framework across different clinical scenarios. The observed findings should therefore be considered exploratory and hypothesis-generating rather than definitive evidence supporting the adoption of the revised criteria. Larger multi-institutional cohorts will be required to validate the reproducibility of the proposed framework. Also notable is that this was a surgical cohort only, so we cannot comment on the usefulness of such revised MPLC criteria for patients with lung cancer undergoing other treatment types.

A further methodological limitation is the restriction of the analysis to the first two resected tumors in patients undergoing three or more surgical resections. Although this decision was made to ensure a consistent and clinically relevant comparison across patients, it may have introduced selection bias by excluding later tumors that could reflect additional biological heterogeneity or disease evolution. Consequently, the analysis may underestimate intrapatient variability and the full spectrum of tumor behavior over time. This restriction also limits generalizability to patients with more complex disease courses involving multiple metachronous lesions. Future studies expanding MPLC vs. IPM classification should be inclusive of third (and subsequent) tumors to better understand the true extent of tumor heterogeneity within individual patients.

An additional limitation relates to the assessment of GGO. GGO classification and the evaluation of the visibility of the second lesion on initial CT/PET imaging were based solely on local radiology reports and were not confirmed by central image re-review. As a result, the classification of GGO status may be subject to interobserver variability and differences in reporting practices across radiologists. This approach may also introduce retrospective assessment bias inherent in the reliance on original clinical reports rather than standardized image re-evaluation. Future studies incorporating standardized, blinded radiologic re-evaluation or quantitative imaging approaches may improve the robustness of GGO-based criteria in distinguishing MPLC from IPM.

A major limitation of the present study is the lack of comprehensive molecular clonality assessment. Limited clinically indicated hotspot mutation panel testing was available for a subset of more recently diagnosed patients; however, these data were not available across the entire cohort. Because molecular testing was performed selectively in more recent cases rather than systematically, the incorporation of these results into the analysis would have introduced potential selection bias and was therefore not undertaken. Systematic next-generation sequencing or whole-exome sequencing data were not available across the cohort and therefore could not be incorporated into the diagnostic framework. Consequently, the proposed revised criteria should not be interpreted as a validated substitute for molecular clonality assessment. Rather, they are intended as a clinically practical framework that leverages routinely available radiographic and pathologic information in settings where comprehensive genomic profiling is unavailable. In this context, our framework is designed to operationalize clinicopathologic and radiographic features that are routinely available in clinical practice while aligning with broader IASLC principles emphasizing multidisciplinary assessment. Future studies incorporating standardized molecular profiling will be necessary to validate, refine, and potentially recalibrate these criteria against genomic measures of tumor clonality. Moving forward, it will be increasingly important to integrate clinical data with molecular assessment to distinguish MPLC from IPM. In their seminal paper, Liu et al., described several patients with tumors that, despite meeting classic criteria for IPM, were observed to have distinct mutational profiles [22]. While these findings are supportive of the need to incorporate tumor heterogeneity in clinical settings, currently, most clinical mutation testing is reserved for actionable mutations; more in-depth testing, such as whole exome sequencing, is rarely performed [23]. To complicate matters further, more comprehensive mutational profiling alone may not be sufficient to differentiate MPLC from IPM; instead, emphasis must be placed on trunk (initiating) vs. branching mutations, as only the discordance of trunk drivers should be indicative of MPLC [16]. How best to incorporate radiographic imaging, clinicopathologic data, and genetic testing towards creating a “gold standard” diagnostic criterion for MPLCs is urgently warranted given the ongoing complexity, controversy, and lack of consensus across research institutes, and this remains an ongoing topic for debate.

One aspect to discuss is the absence of a universally accepted reference standard for definitively distinguishing MPLCs from IPM. As such, our analysis was focused on a comparative assessment of established and exploratory revised classification criteria rather than validation against a biological ground truth. External validation in larger multicenter cohorts and integration with molecular profiling approaches will be essential to confirm the validity, reproducibility, generalizability, and incremental clinical value of the exploratory framework suggested here for more accurately distinguishing MPLC from IPM. The proposed clinicopathologic framework provides a practical and routinely applicable approach for diagnosing MPLCs in settings where molecular clonality assessment is not readily available. However, molecular clonality testing remains among the most biologically robust methods for establishing tumor relatedness and therefore represents a key reference standard. Accordingly, the present framework should be viewed as complementary to, rather than a replacement for, molecular profiling approaches. Our findings raise important questions about the utility of clinicopathologic features for distinguishing MPLC and IPM, but ours or other reclassification frameworks utilizing such variables will require molecular validation or another independent reference standard before being adopted as validated diagnostic criteria.

In conclusion, given these shifts in clinicopathological features, the technological advancements over the past half a century, and the improved understanding of tumor biology, all of which have increased lung cancer survivorship and, subsequently, numbers of individuals expected to have multiple lung cancer diagnoses in their lifetimes, revisions to the Martini and Melamed criteria are needed. However, one of the reasons the Martini and Melamed criteria are so enduring is their simplicity of use, relying on clinical data that is readily available. Advanced histologic, radiologic, and molecular assessment, while promising, may remain out of reach for many patients and could worsen already existing gaps in patient care. The criteria presented here, while unvalidated, suggest that modified criteria utilizing readily available clinicopathological data may be useful for diagnosing MPLC, allowing for more optimized lung cancer care. We hope that the exploratory clinicopathologic reclassification presented here can be validated in the future as part of more comprehensive diagnostic criteria alongside molecular features.

## Figures and Tables

**Table 1:** Demographic and Clinical Descriptions of Patients with Multiple Primary Lung Cancers (MPLCs).

Variable	Original Martini and Melamed Criteria MPLCs, n = 83 (91%)	Preliminary Clinicopathologic Reclassification Framework, MPLC Patients, n = 53 (58%)	Martini and Melamed’s Reported MPLCs, n = 50
Timeframe	Synchronous: 38 (46%) Metachronous: 45 (54%) Mean time between first and second cancer: 30 months	Synchronous: 13 (25%) Metachronous: 40 (75%) Mean time between first and second cancer: 40 months	Synchronous: 18 (36%) Metachronous: 32 (64%) Mean time between first and second cancer: NR
Sex	Male: 31 (37%) Female: 52 (63%)	Male: 18 (34%) Female: 35 (66%)	Male: 38 (76%) Female: 12 (24%)
Age at first lung cancer	Mean: 67 years	Mean: 67 years	Mean: 62 years
Histology of first lung cancer	Squamous: 6 (7%) Adenocarcinoma: 73 (88%) Other 4 (5%) Similar histology between first and second cancer: 71 (86%)	Squamous: 5 (9%) Adenocarcinoma: 45 (85%) Other: 3 (6%) Similar histology between first and second cancer: 41 (77%)	Squamous: 38 (76%) Adenocarcinoma: 5 (10%) Other: 7 (14%) Similar histology between first and second cancer: 33 (66%)
Site of first lung cancer	LLL: 10 (12%) LUL: 20 (24%) RLL: 18 (21%) ** RML: 7 (9%) RUL: 28 (34%) Same lobe both cancers: 10 (12%)	LLL: 6 (11%) LUL: 9 (17%) RLL: 14 (26%) * RML: 3 (6%) RUL: 21 (40%) Same lobe both cancers: 12 (23%)	LLL: NR LUL: NR RLL: NR RML: NR RUL: NR Same lobe both cancers: 10 (20%)
Nodule Size (mean, cm)	N1: 1.96 N2: 1.84	N1: 1.91 N2: 1.62	N1: NR N2: NR
GGO, second nodule	32 (43%)	32 (60%)	NR
Features of intrapulmonary metastasis present	Visceral Pleural Invasion: 14 (17%) *** Vascular Invasion: 20 (24%) Lymphatic Invasion: 30 (36%) Lymph Node Positivity: 1 (1%) Any reported: 36 (43%)	Visceral Pleural Invasion: 6 (11%) Vascular Invasion: 7 (13%) Lymphatic Invasion: 13 (25%) Lymph Node Positivity: 1 (2%) Any reported: 14 (26%)	Visceral Pleural Invasion: 6 (12%) Vascular Invasion: NR Lymphatic Invasion: NR Lymph Node Positivity: 6 (12%) Any reported: NR
Extrapulmonary metastases	Yes: 1 (1%) ****	Yes: 0 (0%)	Yes: 0 (0%)
Surgical treatment of first lung cancer	Pneumonectomy: 0 (0%) Lobectomy: 28 (34%) Sublobar: 55 (66%) Segmentectomy: 3 (4%) Wedge: 52 (63%) Bilateral thoracotomy: 0 (0%) No surgical treatment: 0 (0%)	Pneumonectomy: 0 (0%) Lobectomy: 16 (30%) Sublobar: 37 (70%) Segmentectomy: 1 (2%) Wedge: 36 (68%) Bilateral thoracotomy: 0 (0%) No surgical treatment: 0 (0%)	Pneumonectomy: 13 (26%) Lobectomy: 29 (58%) Sublobar: 2 (4%) Segmentectomy: NR Wedge: NR Bilateral thoracotomy: 3 (6%) No surgical treatment: 3 (6%)

UL = Right Upper Lobe; RML = Right Middle Lobes; RLL = Right Lower Lobe, LUL = Left Upper Lobe; LLL = Left Lower Lobe; NR = not reported; N1 = nodule 1, treated during patient’s first surgery; N2 = nodule 2, treated during patient’s second surgery (or autopsy for Martini and Melamed). Nodule size N2 missing for 3 individuals. NR = Not Reported. * for two patients, two separate tumors observed at the first cancer surgery: one RLL + RML; one RLL + RUL. ** for three patients, two separate tumors observed at the first cancer surgery: RLL + RUL. *** two patients did not have data, percentage calculated with 81 patients. **** one patient did not have data, percentage calculated with 82 patients.

**Table 2:** Reclassification of MPLC vs. IPM under Proposed vs. Original Martini and Melamed Criteria.

		Preliminary Reclassification Framework		
		IPM	MPLC	
Original Martini and Melamed Criteria	IPM	5	3	8 (proportion: 8.8%; 95% CI: 3.9–16.6%)
	MPLC	33	50	83 (proportion: 91.2%; 95% CI: 83.4–96.1%)
		38 (proportion: 41.8%; 95% CI: 31.5–52.6%)	53 (proportion: 58.2%; 95% CI: 47.4–68.5%)	

CI = Confidence Interval.

## Data Availability

The datasets generated and/or analyzed during the current study are available from the corresponding author on reasonable request. Data sharing is subject to institutional regulations and applicable privacy or ethical restrictions.

## Supplementary Materials

The following supporting information can be downloaded at: https://www.mdpi.com/article/10.3390/cancers18142284/s1. Supplementary Table S1: Demographic and Clinical Characteristics of All Patients Treated with More Than One Lung Cancer Surgery (MPLC or IPM), n = 91. Supplementary Table S2: Characteristics of Patients Reclassified under Original Martini and Melamed vs. Exploratory Revised Criteria. Supplementary Figure S1: Exploratory Clinicopathologic Reclassification Framework Compared to Martini and Melamed’s Original Criteria for Diagnosing MPLC vs. IPM. Supplementary Figure S2: Overall Survival (Days) by MPLC Status (Original Diagnostic Criteria; MPLC = 83, IPM = 8). Supplementary Figure S3: Overall Survival (Days) by MPLC Status (Revised Diagnostic Criteria; MPLC = 53, IPM = 38). Supplementary Figure S4: Overall Survival (Days) by Specific Components of the Revised Diagnostic Criteria, from First Surgery.

## Abbreviations

AJCC	American Joint Committee on Cancer
EMR	Electronic Medical Record
GGO	Ground Glass Opacity
LDCT	Low-dose Computed Tomography
IPM	Intrapulmonary Metastasis
IASLC	International Association for the Study of Lung Cancer
MPLCs	Multiple Primary Lung Cancer
NSCLC	Non-small Cell Lung Cancer
UICC	Union for International Cancer Control
VATS	Video-assisted Thoracic Surgery
