# Supplementary material for: Multiple Primary Lung Cancers in Surgical Patients: Revisiting Martini and Melamed 50 Years Later
Source: Cancers (Basel). 2026 Jul 16;18(14):2284. doi: 10.3390/cancers18142284 (PMC13406777; doi:10.3390/cancers18142284)
Supplement: Supplementary file 1 [file cancers-18-02284-s001.zip › cancers-4385360-supplementary.pdf]

Supplementary Material:

Supplementary Table S1: Demographic and Clinical Characteristics of All Patients Treated with More Than One Lung Cancer Surgery (MPLC or IPM), n=91

**Variable**	All patients
**Timeframe**	Synchronous: 46 (51%)<br>Metachronous: 33 (36%)<br><br>Mean time between first and second cancer: 29 months
**Sex**	Male: 33 (36%)<br>Female: 58 (64%)
**Age at first lung cancer**	Mean: 67 years
**Histology of first lung cancer**	Squamous: 8 (9%)<br>Adenocarcinoma: 78 (86%)<br>Other: 5 (5%)<br><br>Similar histology between first and second cancer: 79 (87%)
**Site of first lung cancer**	LLL: 12 (13%)<br>LUL: 21 (23%)<br>RLL: 20 (22%)*<br>RML: 7 (8%)<br>RUL: 31 (34%)<br><br>Same lobe both cancers: 16 (18%)
**Nodule Size (mean, cm)**	N1: 2.02<br>N2: 1.81
**GGO, second nodule**	41 (45%)
**Features of intrapulmonary metastasis present**	Visceral Pleural Invasion: 16 (18%)<br>Vascular Invasion: 22 (24%)<br>Lymphatic Invasion: 33 (36%)<br>Lymph Node Positivity: 3 (3%)<br><br>Any reported: 42 (46%)
**Surgical treatment of first lung cancer**	Pneumonectomy: 0 (0%)<br>Lobectomy: 31 (34%)<br>Sublobar: 60 (66%)<br>Bilateral thoracotomy: 0 (0%)<br>No surgical treatment: 0 (0%)

* for four patients, two separate tumors observed at the first cancer surgery: one RLL + RML; three RLL + RUL

N1 = nodule 1, treated during patient's first surgery; N2 = nodule 2, treated during patient's second surgery. Nodule size N2 missing for 3 individuals

Supplementary Figure S1: Exploratory Clinicopathologic Reclassification Framework Compared to Martini and Melamed's Original Criteria for Diagnosing MPLC vs IPM

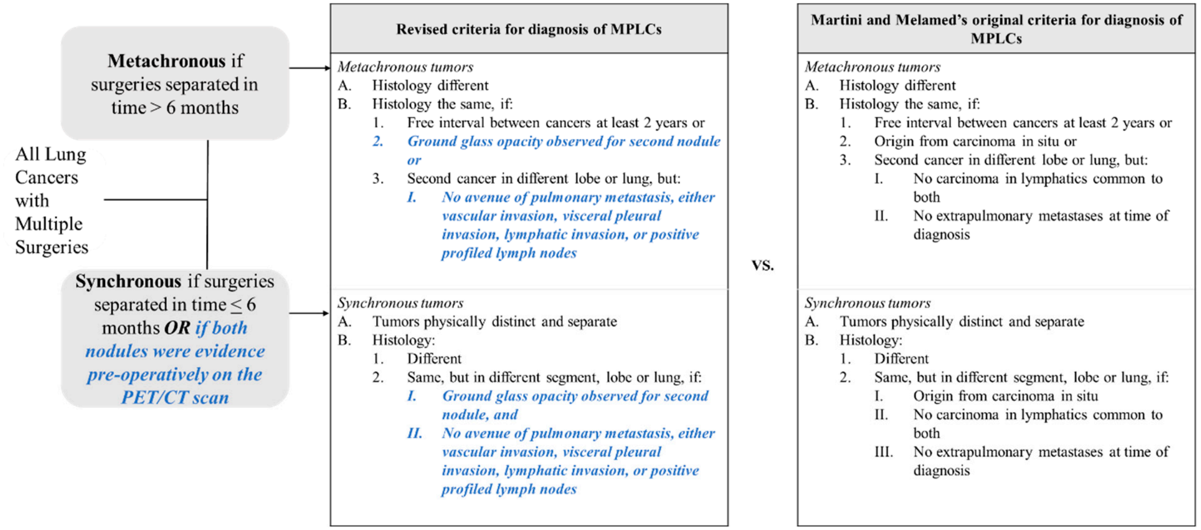

**Blue text is indictive of revisions to their original criteria for distinguishing MPLC from IPM*

Supplementary Table S2: Characteristics of Patients Reclassified under Original Martini and Melamed vs Exploratory Revised Criteria

Patient	MPLC Original Definition	MPLC Revised Definition	Synchronous (<= 6 months)	Metachronous (>= 2 years)	Synchronous (<= 6 months OR both on initial PET/CT Scan)	Visceral Pleural Invasion N1	Vascular Invasion N1	Lymphatic Invasion N1	Positive Lymph nodes N1	Extra Pulmonary Metastases N1	GGO N2
35	No	Yes	No	No	No	No	No	No	No	No	Yes
36	No	Yes	No	No	No	No	No	Yes	No	No	Yes
34	No	Yes	No	No	No	No	No	No	No	No	Yes
4	Yes	No	Yes	No	Yes	Yes	No	No	No	No	No
1	Yes	No	Yes	No	Yes	No	No	Yes	No	No	No
27	Yes	No	Yes	No	Yes	No	Yes	Yes	No	Yes	No
3	Yes	No	Yes	No	Yes	No	Yes	Yes	No	Yes	Yes
18	Yes	No	Yes	No	Yes	No	No	No	No	No	No
16	Yes	No	No	No	Yes	No	Yes	Yes	No	Yes	Yes
26	Yes	No	No	Yes	Yes	No	Yes	No	No	Yes	Yes
14	Yes	No	No	No	Yes	No	No	No	No	No	No
15	Yes	No	Yes	No	Yes	Yes	Yes	Yes	No	Yes	No
33	Yes	No	Yes	No	Yes	Yes	Yes	Yes	No	Yes	N/A
9	Yes	No	Yes	No	Yes	Yes	No	Yes	No	No	No
7	Yes	No	Yes	No	Yes	No	No	No	No	No	No
5	Yes	No	No	No	Yes	No	Yes	No	No	Yes	No
30	Yes	No	Yes	No	Yes	No	Yes	Yes	No	Yes	Yes
29	Yes	No	No	No	Yes	Yes	Yes	Yes	No	Yes	No
28	Yes	No	Yes	No	Yes	Yes	No	No	No	No	Yes
25	Yes	No	No	Yes	Yes	Yes	No	Yes	No	No	No

22	Yes	No	No	No	Yes	No	No	Yes	No	No	Yes
21	Yes	No	Yes	No	Yes	No	No	No	No	No	No
23	Yes	No	Yes	No	Yes	No	No	No	No	No	N/A
31	Yes	No	Yes	No	Yes	Yes	No	Yes	No	No	No
20	Yes	No	No	No	Yes	No	No	No	No	No	No
10	Yes	No	No	Yes	Yes	No	No	Yes	No	No	Yes
17	Yes	No	No	No	Yes	No	Yes	Yes	No	No	No
6	Yes	No	No	Yes	Yes	No	No	No	No	Yes	No
11	Yes	No	Yes	No	Yes	No	No	No	No	No	No
13	Yes	No	No	Yes	Yes	No	No	No	No	No	No
12	Yes	No	Yes	No	Yes	No	Yes	Yes	No	No	Yes
19	Yes	No	Yes	No	Yes	No	No	Yes	No	No	Yes
8	Yes	No	No	No	Yes	No	Yes	No	No	No	No
24	Yes	No	No	No	No	No	Yes	Yes	No	No	No
32	Yes	No	Yes	No	Yes	No	No	Yes	No	No	No
2	Yes	No	No	No	Yes	No	No	No	No	No	No

*N1 = nodule 1, treated during patient's first surgery; N2 = nodule 2, treated during patient's second surgery.

Clinical information of all patients who met original criteria for MLPC, but did not meet revised criteria for MPLC. First three rows are patients who did not meet the original criteria for MPLC, but did meet revised criteria for MPLC.

Supplementary Figure S2: Overall Survival (Days) by MPLC Status (Original Diagnostic Criteria; MPLC = 83, IPM = 8)

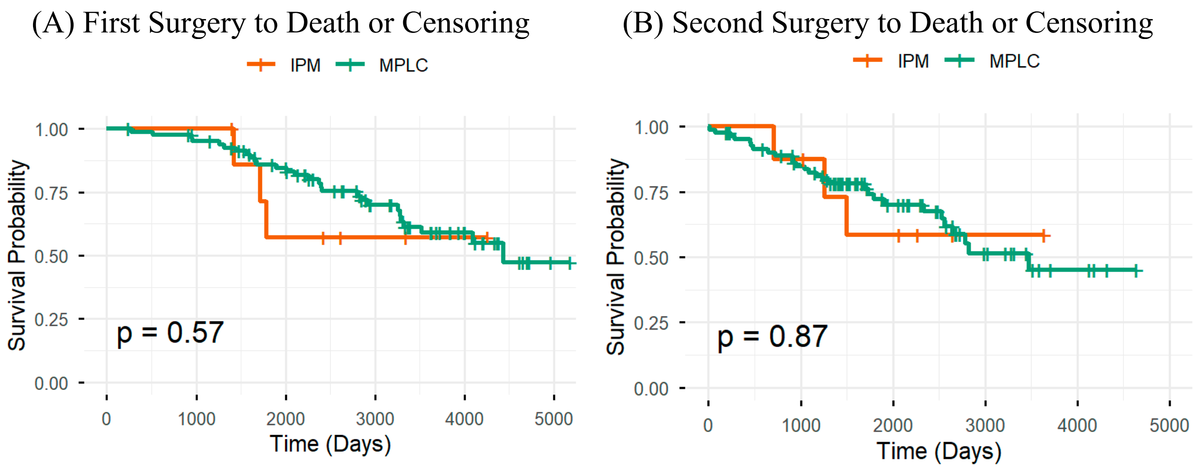

HR$_{\text{first surgery}}$: 0.74; 95% CI: 0.21-2.33

HR$_{\text{second surgery}}$: 0.90; 95% CI: 0.27-2.98

Supplementary Figure S3: Overall Survival (Days) by MPLC Status (Revised Diagnostic Criteria; MPLC = 53, IPM = 38)

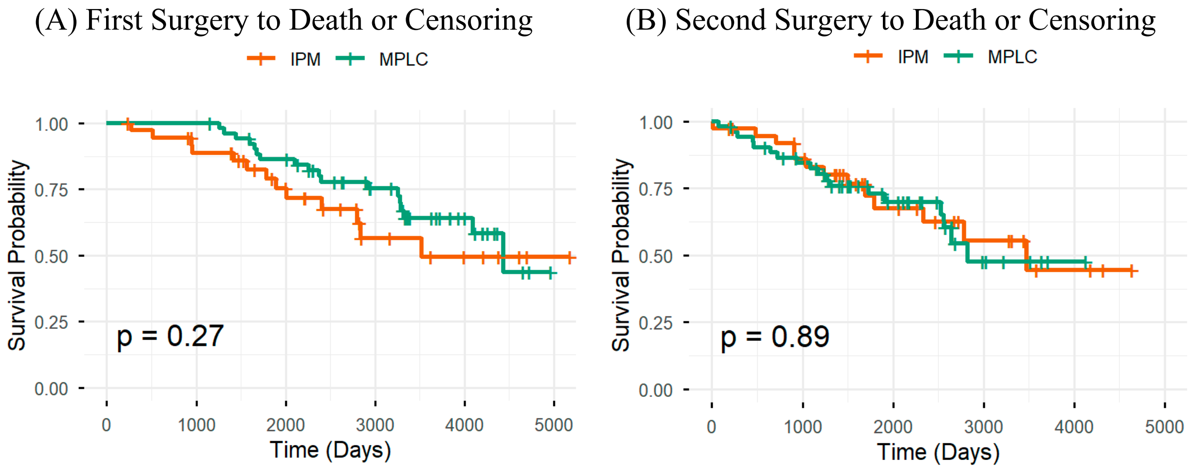

HR$_{\text{first surgery}}$: 0.67; 95% CI: 0.33-1.37

HR$_{\text{second surgery}}$: 1.05; 95% CI: 0.51-2.15

Supplementary Figure S4: Overall Survival (Days) by Specific Components of the Revised Diagnostic Criteria, from First Surgery

(A) Any Pathway of Metastatic Invasion (Lymphatic, Visceral Pleural, Vascular or Lymph Node Invasion) (n=44/91)

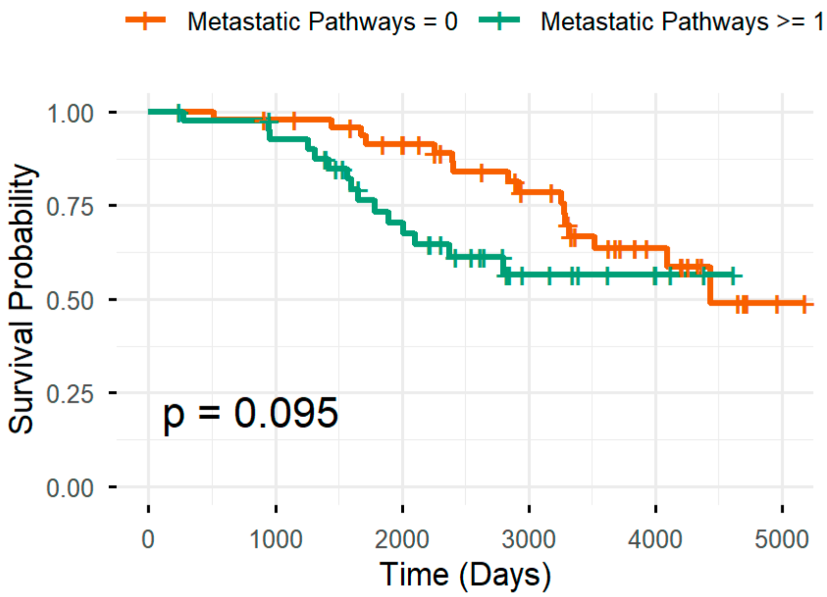

$HR_{first\ surgery}$: 1.83; 95% CI: 0.89-3.76

(B) Lymphatic Invasion (n=33/91)

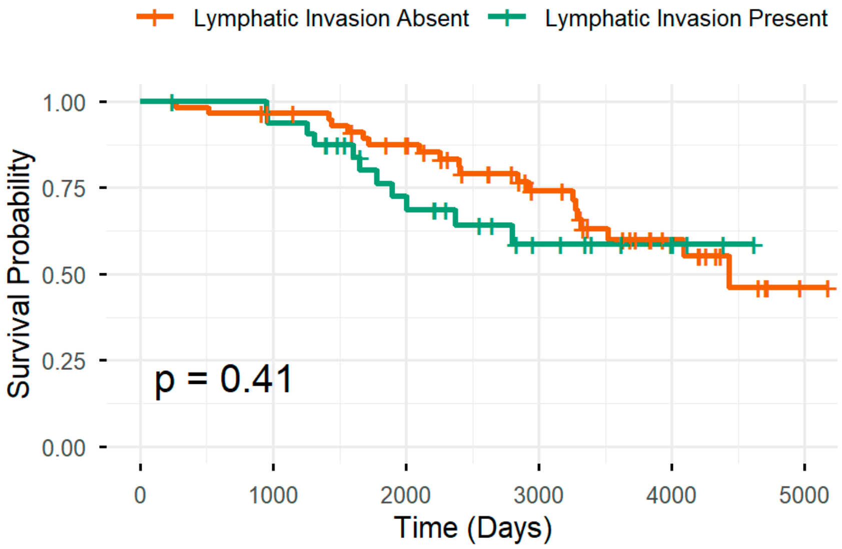

$HR_{first\ surgery}$: 1.36; 95% CI: 0.65-2.86

(C) Visceral Pleural Invasion (n=16/91)

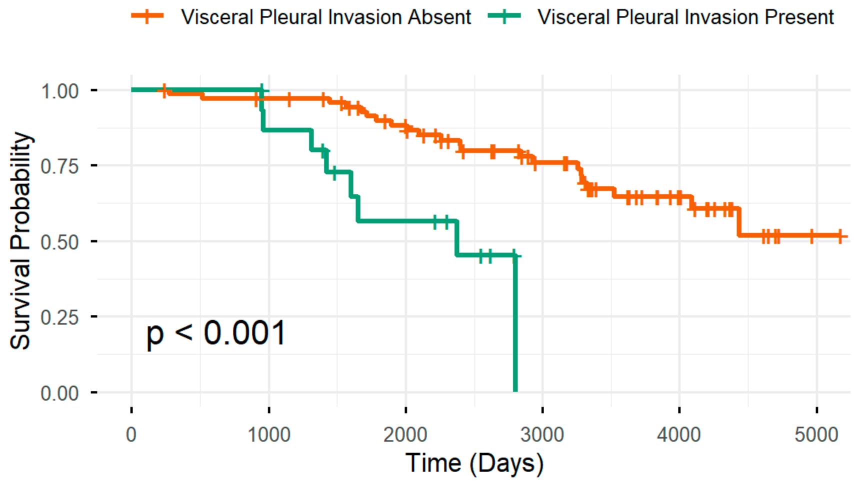

$HR_{first\ surgery}$: 4.67; 95% CI: 1.91-11.47

(D) Vascular Invasion (n=22/91)

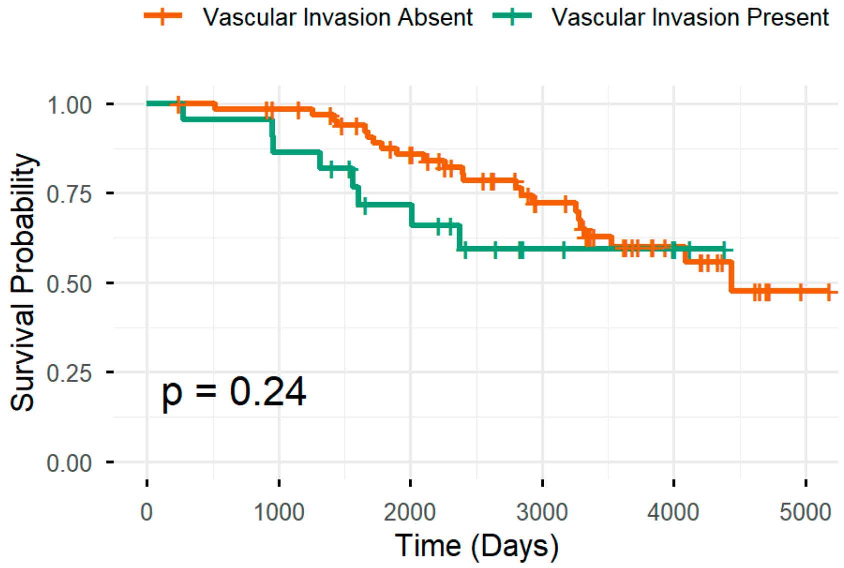

$HR_{first\ surgery}$: 1.62; 95% CI: 0.72-3.67

(E) Lymph Node Positivity (n = 3/91)

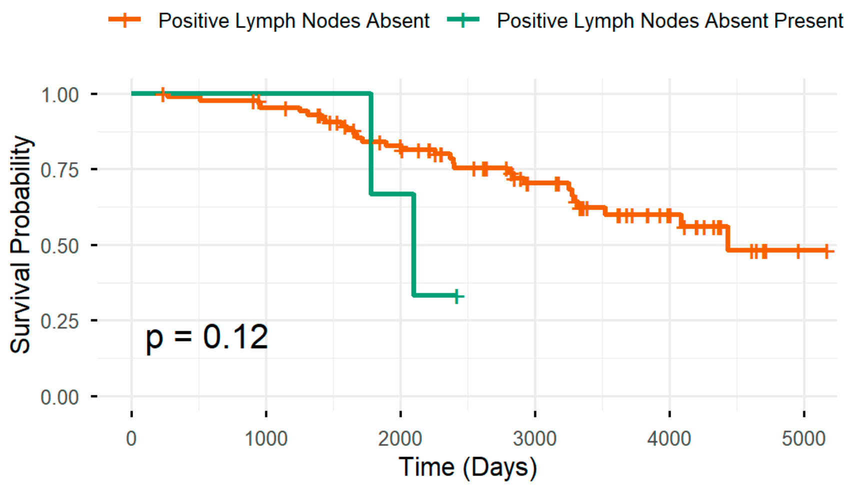

HR$_{first\ surgery}$: 3.03; 95% CI: 0.70-13.06

(F) Number of Potential Pathways

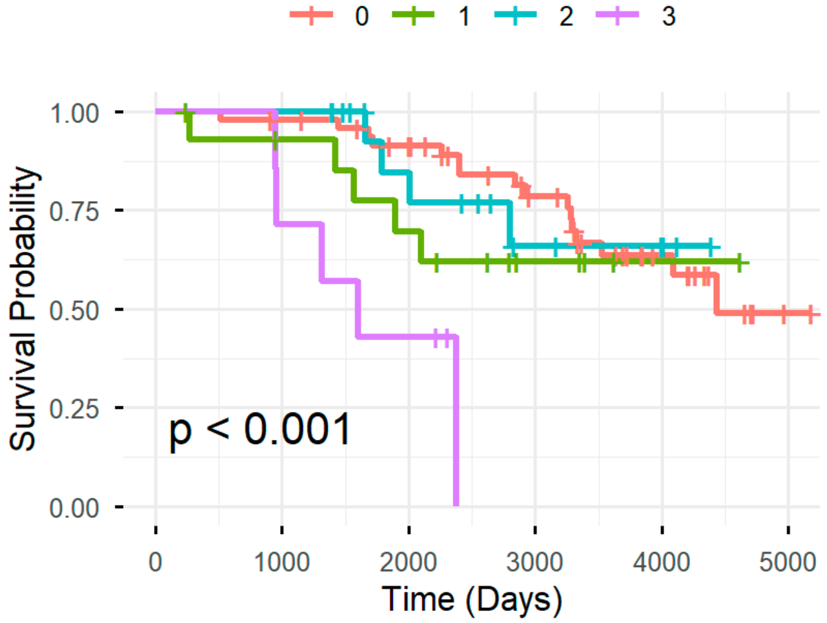

HR$_{first\ surgery}$: 1.52; 95% CI: 1.06-2.18
